# Supplementary material for: Anti-Inflammatory Activity of Mulberry Leaf Flavonoids In Vitro and In Vivo
Source: Int J Mol Sci. 2022 Jul 12;23(14):7694. doi: 10.3390/ijms23147694 (PMC9318041; doi:10.3390/ijms23147694)
Supplement: Supplementary file 1 [file ijms-23-07694-s001.zip › Table S1.pdf]

Table S1. Putatively identified substances among MLFs based on a metabolomics analysis.

Compound name	Retention time/min	Detected mass（ESI+）	Ion types	MS/MS fragments
(Z)-Resveratrol 4'-glucoside	9.27	391.1396	M+H	229, 149, 135
Aesculin 1	7.68	341.0868	M+H	179, 133, 123
Aesculin 2	7.12	341.088	M+H	179, 133, 123
Chlorogenic acid 1	8.45	355.1028	M+H	163, 145, 117, 89
Cis-Mulberroside A	8.73	569.1868	M+H	407, 245, 227, 181, 135
Butin	21.76	273.0755	M+H	137, 81
Loureirin B	20.88	317.1382	M+H	299, 167, 149, 121
Kaempferol[a]	22.32	287.0548	M+H	287, 153, 135, 107
Kaempferol 3-(6''-malonylglucoside)	22.88	535.1085	M+H	287, 159, 135
Kaempferol 3-O-diglucoside 1	13.95	611.1607	M+H	449, 287
Kaempferol 3-O-diglucoside 2	9.82	611.1614	M+H	449, 287
Kaempferol 3-O-diglucoside 3	13.03	611.1622	M+H	449, 287
Kaempferol 3-O-diglucoside 4	16.31	611.1623	M+H	449, 287
Kaempferol 3-O-diglucoside 5	15.42	633.1445	M+Na	633, 347
Kaempferol 3-O-diglucoside isomer 1	10.27	611.1606	M+H	449, 287
Kaempferol 3-O-diglucoside isomer 2	10.84	611.1616	M+H	449, 287
Kaempferol 3-O-diglucoside isomer 3	8.68	611.1622	M+H	449, 287
Kaempferol 3-O-diglucoside isomer 4	13.14	611.1626	M+H	449, 287
Kaempferol 3-O-dirhamnosylglucoside	17.66	741.2248	M+H	595, 449, 287
Kaempferol 3-O-rutinoside 1	21.82	595.1658	M+H	449, 287
Kaempferol 3-O-rhamnosyldiglucoside	12.3	795.1751	M+H	644
Kaempferol 3-O-rhamnosyldiglucoside isomer 1	8.644	757.2204	M+H	611, 449, 287
Kaempferol 3-O-rhamnosyldiglucoside isomer 2	10.58	757.2208	M+H	611, 449, 287
Kaempferol-3-O-glucoside[a]	17.47	449.1077	M+H	287
Kaempferol glucoside 1	21.54	449.1082	M+H	287
Kaempferol glucoside 2	9.24	449.1085	M+H	287
Quercetin[a]	14.65	303.0496	M+H	257, 229, 165, 153, 137
Quercetin 3-(6''-malonyl-glucoside)	19.95	551.1037	M+H	303, 159, 127
Quercetin 3-O-dirhamnosylglucoside 1	12.71	757.2185	M+H	611, 465, 303
Quercetin 3-O-dirhamnosylglucoside 2	15.42	757.2191	M+H	611, 465, 303
Quercetin 3-O-dirhamnosylglucoside 3	9.85	757.2192	M+H	611, 465, 303
Quercetin 3-O-dirhamnosylglucoside 4	16.04	757.2195	M+H	611, 465, 303
Quercetin 3-O-dirhamnosylglucoside isomer 1	12.28	757.219	M+H	611, 465, 303
Quercetin 3-O-dirhamnosylglucoside isomer 2	11.76	757.2207	M+H	611, 465, 303
Quercetin-3-O-rutinoside isomer 1	12.57	611.1608	M+H	611, 465, 303

Quercetin-3-O-rutinoside isomer 2	14.99	611.1614	M+H	465, 303
Quercetin-3-O-rutinoside isomer 3	11.73	611.1621	M+H	465, 303
Quercetin 3-O-rhamnosyldiglucoside 1	13.56	773.214	M+H	627, 465, 303
Quercetin 3-O-rhamnosyldiglucoside 2	10.76	773.2143	M+H	627, 465, 303
Quercetin 3-O-rhamnosyldiglucoside 3	9.07	773.2145	M+H	627, 465, 303
Quercetin-3-O-glucoside[a]	10.84	465.1026	M+H	303, 153, 149
Quercetin glucoside 1	12.53	465.103	M+H	303
Quercetin glucoside 2	18.62	465.103	M+H	303
Quercetin glucoside 3	14.11	465.1035	M+H	303
Quercetin rutinoside	18.87	611.161	M+H	465, 303
Quercetin-diglucoside 1	10.42	627.1562	M+H	465, 303
Quercetin-diglucoside 2	13.71	627.1563	M+H	465, 303
Quercetin-diglucoside 3	9.58	627.1564	M+H	465, 303
Quercetin-diglucoside 4	8.86	627.1575	M+H	465, 303
Quercetin-diglucoside 5	14.07	649.1391	M+Na	649, 347

[a] Confirmed by the authentic standards.
