# Supplementary material for: Anti-Inflammatory Activity of Mulberry Leaf Flavonoids In Vitro and In Vivo
Source: Int J Mol Sci. 2022 Jul 12;23(14):7694. doi: 10.3390/ijms23147694 (PMC9318041; doi:10.3390/ijms23147694)
Supplement: Supplementary file 1 [file ijms-23-07694-s001.zip › Table S2 .pdf]

Table S2. Standard for Evaluation of the Disease Activity Index (DAI)[a,b,c]

Weight loss (%)	Stool consistency	Blood	Score
0	Normal stools	Negative hemoccult (no bleeding)	0
1-5	Loose stools but still formed	Negative hemoccult	1
5-10	Loose stools	Positive hemoccult (slight)	2
10-15	Darrhea (slight)	Positive hemoccult	3
>15	Watery diarrhea	Gross bleeding	4

[a]DAI is calculated as the sum of weight loss, stool consistency and hematochezia.
[b]Body weight loss is the percentage difference between the initial body weight (day 0) and the body weight on any particular day.
[c] Normal stools: well-formed stool; loose stools: pasty stool that does not stick to the anus; diarrhea: liquid stools that adheres to the anus.
