# Supplementary material for: Anti-Inflammatory Activity of Mulberry Leaf Flavonoids In Vitro and In Vivo
Source: Int J Mol Sci. 2022 Jul 12;23(14):7694. doi: 10.3390/ijms23147694 (PMC9318041; doi:10.3390/ijms23147694)
Supplement: Supplementary file 1 [file ijms-23-07694-s001.zip › Table S3.pdf]

Table S3. Standard for Evaluation of Histological Injury.

Feature	Description	Score
Inflammation	Normal	0
	Mild	1
	Moderate	2
	Severe	3
Mucosal injury	Normal	0
	Mucous layer	1
	Submucosa	2
	Muscularis and serosa	3
Glands	Normal	0
	Minimal: rare gland dilatation	1
	Mild: multifocal gland dilatation	2
	Moderate: multifocal gland dilatation with abscessation and occasional loss of glands	3
Area of section affected	None	0
	<10%	1
	10–25%	2
	26–50%	3
	>50%	4
